# Rapid song divergence leads to discordance between genetic distance and phenotypic characters important in reproductive isolation

**DOI:** 10.1002/ece3.3673

**Published:** 2017-12-05

**Authors:** Emmanuel C. Nwankwo, Chryso Th. Pallari, Louis Hadjioannou, Andreas Ioannou, Ronald K. Mulwa, Alexander N. G. Kirschel

**Affiliations:** ^1^ Department of Biological Sciences University of Cyprus Nicosia Cyprus; ^2^ Ornithology Section, Zoology Department National Museums of Kenya Nairobi Kenya; ^3^ Department of Zoology Edward Grey Institute University of Oxford Oxford UK

**Keywords:** birds, molecular phylogenetics, morphology, playback experiments, plumage coloration, *Pogoniulus*, species limits, tinkerbirds, vocalizations

## Abstract

The criteria for species delimitation in birds have long been debated, and several recent studies have proposed new methods for such delimitation. On one side, there is a large consensus of investigators who believe that the only evidence that can be used to delimit species is molecular phylogenetics, and with increasing numbers of markers to gain better support, whereas on the other, there are investigators adopting alternative approaches based largely on phenotypic differences, including in morphology and communication signals. Yet, these methods have little to say about rapid differentiation in specific traits shown to be important in reproductive isolation. Here, we examine variation in phenotypic (morphology, plumage, and song) and genotypic (mitochondrial and nuclear DNA) traits among populations of yellow‐rumped tinkerbird *Pogoniulus bilineatus* in East Africa. Strikingly, song divergence between the *P. b. fischeri* subspecies from Kenya and Zanzibar and *P. b. bilineatus* from Tanzania is discordant with genetic distance, having occurred over a short time frame, and playback experiments show that adjacent populations of *P. b. bilineatus* and *P. b. fischeri* do not recognize one another's songs. While such rapid divergence might suggest a founder effect following invasion of Zanzibar, molecular evidence suggests otherwise, with insular *P. b. fischeri* nested within mainland *P. b. fischeri*. Populations from the Eastern Arc Mountains are genetically more distant, yet share the same song with *P. b. bilineatus* from Coastal Tanzania and Southern Africa, suggesting they would interbreed. We believe investigators ought to examine potentially rapid divergence in traits important in species recognition and sexual selection when delimiting species, rather than relying entirely on arbitrary quantitative characters or molecular markers.

## 1 INTRODUCTION

Historically, species delimitation was based on morphological differences identified among specimens of organisms by natural historians and collectors (Agardh, 1852; Linnaeus, 1758). Over time, several alternative species concepts had been proposed, and Ernst Mayr's Biological Species Concept (BSC), focusing on the ability of different groups to interbreed (Mayr, 1963), gained much favor. However, because related groups of organisms are commonly distributed allopatrically, delimitation of species using the BSC has seldom been tested in nature (Lagache, Leger, Daudin, Petit, & Vacher, 2013). Because species diversification results from cumulative changes in heritable traits among populations, molecular phylogenetics have become the most commonly used methodology for species delimitation, with phylogenies reflecting evolutionary histories, and accordingly, the phylogenetic species concept (PSC) has prevailed in recent times, especially in studies on birds (Barrowclough, Cracraft, Klicka, & Zink, 2016; Cracraft, 1992). Fine‐scale phylogeographic studies have used one or more genes, often mitochondrial genes, to establish patterns of historical biogeography. But, mitochondrial gene trees are not always congruent with species trees (Edwards et al., 2005; Moore, 1995; Rokas, Williams, King, & Carroll, 2003), often because of mito‐nuclear discordance (Toews & Brelsford, 2012). More recent studies have incorporated nuclear genes to determine the extent of concordance with faster‐evolving mitochondrial genes, or even orders of magnitude more markers via next‐generation sequencing methods (NGS) such as double‐digest restriction‐associated DNA sequencing (ddRADseq) and hybrid enrichment (reviewed in Lemmon & Lemmon, 2013).

While phylogeographic studies might reveal to a greater or lesser extent how long populations have been evolving in isolation, they reveal little with regard to which diverging characters lead to reproductive isolation and speciation. Divergence in characters that lead to reproductive isolation could occur at a much faster rate than the mutation rate of many markers used in phylogenetic studies (Mallet, 2005; but see Winger & Bates, 2015), such as in a gene affecting visual or acoustic communication signals that affect mate choice or species recognition. An alternative approach for species delimitation was proposed by Tobias et al. (2010), based on a calibration of phenotypic differences of a set of 58 species pairs occurring in sympatry, and applying that calibration to species in allopatry. The study has stimulated a heated debate between its authors and proponents (e.g., Collar et al., 2016) and PSC supporters (e.g., Remsen, 2015), although neither system addresses the issue of reproductive isolation among species.

Rapid divergence in characters leading to reproductive isolation between populations could be driven by natural selection, by adaptation to differences between their constituent environments (Dynesius & Jansson, 2000), or sexual selection (Gavrilets, 2000), or even by genetic drift resulting from a founder effect. Mayr (1947, 1982) championed the role of drift driving “genetic revolutions” in his definition of peripatric speciation, the process he proposed to explain divergence in phenotypic characters in island populations following dispersal from the mainland. If such rapid speciation can occur in such circumstances, how does it correspond with genetic divergence in a group of related organisms?

In birds, song, color pattern, and morphology are important signals in conspecific interaction (Andersson, 1994; Candolin, 2003; Clayton, 1990; Partan & Marler, 2005). Over time, divergence in these traits across populations can lead to positive assortative mating with corresponding divergence in the recognition of those traits (Price, 1998), and consequently reproductive isolation. Studies using experiments with taxidermic mount presentations and song playback have shown how divergence in phenotypic traits might lead to species recognition failure, including in plumage and song (e.g., Uy, Moyle, Filardi, & Cheviron, 2009). There is also much evidence of increased body size (e.g., gigantism) between island populations and populations on the mainland (Murphy, 1938). Body size is negatively correlated with song frequency in birds (Bertelli & Tubaro, 2002; Ryan & Brenowitz, 1985; Seddon, 2005; Wallschläger, 1980), and bill shape with song pace (Podos, 2001), and divergence in these morphological traits could lead to distinct song differences affecting mate choice or species recognition.

**Figure 1:**
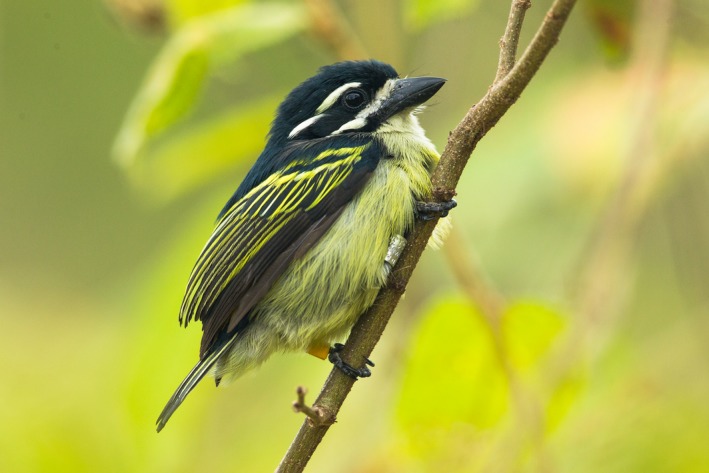
A yellow‐rumped tinkerbird (*Pogoniulus bilineatus*) ringed at Genda Genda, Tanzania

Little is known regarding the extent to which such phenotypic divergence corresponds to genetic divergence among populations with varying extents of genetic isolation. In the present study, we examined the patterns of diversification between populations of yellow‐rumped tinkerbird (*Pogoniulus bilineatus*, Figure 1) with the specific objective of comparing variation in song characteristics and song recognition, morphology, and plumage, and genetic differentiation between populations of the subspecies *P. bilineatus bilineatus* (hereafter *bilineatus*) and *P. bilineatus fischeri* (hereafter *fischeri*) in Eastern to Southern Africa. We focused on these subspecies because they had been reported to emit distinctly different vocalizations (e.g., Stevenson & Fanshawe, 2002). We thus wanted to investigate the possibility that divergence in song could have resulted from a founder effect following dispersal to island populations, and specifically Zanzibar, and compare patterns of phenotypic variation with genetic divergence among populations. The aim of the study was thus to understand the mode of speciation between the two subspecies, and the extent to which phenotypic divergence corresponds with genetic differentiation. In addition to *fischeri* from coastal Kenya and Zanzibar (specifically Unguja island) and *bilineatus* from Coastal Tanzania southwards to eastern South Africa, we also included populations from the lower Eastern Arc Mountains in the study. Some recent studies on other species from these mountains have revealed high levels of differentiation (Bowie & Fjeldså, 2005; Bowie, Fjeldså, Hackett, & Crowe, 2004; Burgess et al., 2007; Fjeldså, Bowie, & Rahbek, 2012). A subspecies of yellow‐rumped tinkerbird *P. b. conciliator* (hereafter *conciliator*) was described from the Uluguru Mountains within the Eastern Arc Mountains by Friedmann (1929), but subsequently synonymized with *fischeri* (Short & Horne, 2002), yet the morphological traits that led Friedmann (1929) to originally describe it as different mean it is worth investigating in the context of differential patterns of phenotypic and genetic divergence among regional populations.

We hypothesized that because song is considered an innate character in Piciformes (Kirschel, Blumstein, & Smith, 2009), variation in song, along with heritable morphological characters, should be concordant with patterns of population genetic structure and phylogeny. We thus examined patterns of song variation in the field and tested the extent to which variation was detectable to different populations using playback experiments, and examined patterns of variation in morphology and plumage in museum specimens, while collecting DNA samples in the field for molecular phylogenetic and population genetic analyses.

## 2 MATERIALS AND METHODS

### 2.1 Field recordings and song analyses

We examined variation in acoustic signal characteristics in 67 yellow‐rumped tinkerbird from East to Southern Africa. Songs were recorded in the field at 25 sites in Kenya and Tanzania, including Zanzibar, between July 2011 and March 2014, and in Swaziland in March 2015, using a Marantz PMD 661 (Marantz Corporation, Kanagawa, Japan) solid‐state digital recorder with a Sennheiser MKH‐8020 microphone housed in a Telinga Universal parabolic reflector, or a Sennheiser MKH‐8050 directional microphone housed in a Rycote Modular Windshield WS 9 Kit, at 16 bits and a 48‐kHz sampling rate. Raven 1.4 software was used for sound analyses (Charif, Strickman, & Waack, 2010). Five songs per recording were measured and mean values calculated. The following parameters were measured: peak frequency (Hz), delta time (s), last pulse duration, internote interval (gap), and number of pulses. The rate of each song was computed using the formula below (from Kirschel, Blumstein, & Smith, 2009):

$$R=\frac{\left(\text{NP}-1\right)}{\left(\text{DT}-\text{LC}\right)}$$

where *R* = rate of song, NP = number of pulses, DT = delta time and LC = last pulse duration.

### 2.2 Playback experiments

For the playback experiments, we used synthetically produced playbacks as stimuli, in order to prevent pseudoreplication associated with authentic recordings (McGregor, Dabelsteen, Shepherd, & Pedersen, 1992). This method permits testing specifically for the effect of differences in frequency and rate between the two subspecies’ songs and avoids motivational, signal‐to‐noise ratio, geographic population, background noise, or other differences inherent in field recordings (McGregor et al., 1992). For the *fischeri *stimulus, we took mean values from 21 recordings collected from four populations in Kenya in 2011/2012. For *bilineatus, *we used 10 recordings collected from 10 populations in Tanzania in 2013. Mean song frequency and rate from all the recordings for each subspecies were used to produce the synthetic playback stimuli using Audacity (Audacity, v. 1.3.3, 2011). Some experiments were performed using an alternative *bilineatus* synthetic stimulus, for example, for experiments in Kenya in 2012 prior to obtaining recordings of *bilineatus* from Tanzania in 2013 in order to produce the stimulus, and in those cases, we used a previously prepared synthetic stimulus for *P. bilineatus* based on recordings from Uganda (Kirschel, Blumstein et al., 2009). The Uganda song‐based stimulus was prepared using a song frequency and rate well within the range of variation of recordings used to prepare the *bilineatus* from Tanzania stimulus.

Playback experiments were performed on 16 individuals (five in Tanzania, eight in Zanzibar, and three in Kenya). Each paired playback experiment lasted 12 min, during which two sets of stimuli were presented. For each individual, we placed a speaker and playback recorder within 30 m of the location of the focal bird. In all cases, distances were estimated to the nearest meter and behavioral data were recorded during the playback experiment. The playback stimuli consisted of 1‐min silence preplayback, 2‐min playback of the first song stimulus, and 1‐min silence postplayback. The focal bird's singing behavior was recorded during the preplayback silence and then during stimulus playback and in the postplayback silence to allow for testing of the effect of playback on song behavior. A further 4 min of silence was observed before initiating the second experiment of the pair. Six variables were selected to characterize behavioral responses (see Table 1). The six variables were reduced to a single variable by factor analysis of mixed data (FAMD, Pagès, 2004; Lê et al., 2008). This method calculated principal components accounting for both continuous and categorical variables. The first principal component was used to test for significant variation in responses to playbacks using a generalized linear model.

**Table 1:** Behavioral variables used to measure response of focal individuals to song playback

Variable	Description
Latency to flight toward speaker (L) (s)	Time until the focal bird flew toward the playback speaker, recorded in seconds
Total time spent (*T*_*t*_) (s)	Total time spent by the bird within 20 m for the entire playback experiment
Number of songs before playback (NS_bp_)	Number of songs emitted by the focal bird before the commencement of the playback (preplayback)
Number of songs during playback (NS_dp_)	Number of songs of the focal bird during the playback
Number of songs after playback (NS_ap_)	Number of songs of the focal bird after the playback (postplayback)
Closest distance	The closest distance of the focal bird to the playback speaker

### 2.3 Song analysis

We used linear mixed models to test for differences in song frequency and rate among populations (Cnaan, Laird, & Slasor, 1997). In order to control for possible variation within regions explained by ecological gradients, we included elevation as a predictor in the model, and site was included as a random effect to control for any variation explained within site locations. The 25 sites were grouped into three populations as follows: *bilineatus* comprising 14 sites in coastal forest in Tanzania and one in Swaziland; *conciliator* comprising the Udzungwa mountains; and *fischeri* comprising seven sites along the coast of Kenya and two in Zanzibar.

Several models were run in R 3.3.0 (R Core Team, 2017) and the best approximating model in explaining variation in song rate and peak frequency was selected using the backward deletion method and compared with the full model using an information‐theoretic approach based on Akaike's information criterion (AIC).

We compared differences in the dispersion of song frequency and rate among populations, using the coefficient of variation, calculated using the formula:

$$CV=\frac{\mathrm{SD}}{\text{Mean}}\times 100$$

where *CV* = coefficient of variation and *SD* = standard deviation.

We tested for homogeneity of variances of the measured song variables across populations as a prerequisite to the use of parametric statistical tests based on Levene's test implemented within R 3.3.0 (Table S1). A multiresponse permutation procedure of within‐ versus among‐group dissimilarities was used to ensure that significant variations observed were not as a result of greater variation in song properties within the populations (vegan, Oksanen, Blanchet, Kindt, Legendre, & O'Hara, 2016). We also used a generalized canonical discriminant analysis on the song properties to estimate the degree of classification efficiency of the populations based on their song characteristics (candisc, Friendly & Fox, 2016).

### 2.4 Morphometrics

Specimens from the ornithology collections of the Natural History Museum of Los Angeles County (LACM), British Museum of Natural History (BMNH), and Zoological Museum of the University of Copenhagen (ZMUC) were used to obtain morphometric data. In total, we took morphometric measurements from 71 specimens. Informed by our findings in the field, we assigned subspecies for analysis accordingly: *fischeri* = 19 specimens from Kenya and Zanzibar, *bilineatus* = 41 specimens from Tanzania coast, Mafia Island, Mozambique, Malawi, Zimbabwe, and South Africa; and *conciliator* = 11 specimens from the Eastern Arc mountains (Uluguru, Nguru, and Udzungwa mountains). Measurements were taken using digital calipers, and included wing chord, tarsus and tail length, bill length and width, and lower mandible length. The localities where specimens were collected were identified from museum tags, maps, gazetteers, and Google Earth (Google, Inc.), for those specimens where coordinates were not given, and assigned approximate latitude and longitude coordinates and elevation for each locality. Moderate Resolution Imaging Radiometer raster files at 250‐m resolution (MODIS/Terra Vegetation Indices 16‐Day L3 Global 250 m) from 2010 were used to extract vegetation continuous field (VCF) and enhanced vegetation index (EVI) data for coordinates of the sampling locations in ArcGIS 10.1 (ESRI, 2012).

Principal component analysis (PCA) was used in the analysis of the morphology data, which allowed us to reduce the eight morphological variables (wing, tarsus, tail, bill length, culmen, upper bill depth, bill width, and lower mandible) into two principal components to determine the dimensions of variability in body size and bill shape of the study species. The PCA was performed in STATA 10.1 (StataCorp, 2009). The first principal component was used to test for variation in morphology resulting from differences between the sexes using a generalized linear mixed‐effects model.

### 2.5 Plumage color measurements

We measured reflectance spectra (200–900 nm) of feathers on the breast, belly, and rump of 31 museum study skins at the BMNH and ZMUC, incorporating 10 *fischeri* (Kenya/Zanzibar), 10 *bilineatus* from Tanzania, three *conciliator* from the Eastern Arc Mountains (Udzungwa), and eight *bilineatus* from Southern Africa, using a JAZ spectrometer (Ocean Optics) with a fiber‐optic reflectance probe (Ocean Optics R‐200) and PX xenon light source. The reflection probe was placed in an RPH‐1 Reflection Probe Holder (Ocean Optics), at a 90**°** angle, and secured at 2 mm from the aperture of the probe holder. Two measurements were taken per plumage patch, per specimen, with the specimen placed flat onto a white background perpendicular to the observer and facing to the left, and then rotated 180**°** for the second measurement, with the probe holder placed horizontally onto the specimen, so the aperture completely covered the feather patch, thus ensuring ambient light was excluded. Reflectance data for each specimen were obtained following calibration with a white standard (Ocean Optics WS‐1) and dark standard (by screwing the lid back onto the fiber‐optic connector to ensure no light entered), and recorded in SPECTRASuite (Version 1.0, Ocean Optics).

### 2.6 Plumage analysis

Pavo (an R package for the perceptual analysis, visualization, and organization of color data, Maia, Eliason, Bitton, Doucet, & Shawkey, 2013) implemented within R statistical software (version 3.2.4) was used for the plumage color analysis. Replicate reflectance spectra were averaged and smoothed with a span of 0.25 for further analysis. Negative value correction on the spectra data was effected by adding min to all reflectance. The color distances within and between the populations were calculated using the function *coldist,* which applies the visual models of Vorobyev, Osorio, Bennett, Marshall, and Cuthill (1998) to calculate color distances with receptor noise based on relative photoreceptor densities. A hue projection plot was produced using the function *projplot*. This is a 2D plot of color points projected from the tetrahedron to its encapsulating sphere to visualize differences in hue. The Mollweide projection was used in the hue projection plot instead of the Robinson projection, because the Mollweide projection preserves area relationships within latitudes without distortion (Maia, et al., 2013). The avian tetracolor space visual model was computed using the *tcs* function, which calculates coordinates and colorimetric variables that represent reflectance spectra in the avian tetrahedral color space: *u, s, m, l* (the quantum catch data); *u.r, s.r, m.r, l.r* (relative cone stimulation, for a given hue, as a function of saturation); *x, y, z* (cartesian coordinates for the points in the tetrahedral color space); *h.theta, h.phi* (angles theta and phi, in radians, determining the hue of the color); *r.vec* (the r vector indicating saturation, distance from the achromatic center); *r.max* (the maximum r vector achievable for the color's hue); *r.achieved* (the relative r distance from the achromatic center, in relation to the maximum distance achievable). The function *voloverlap* was used to calculate the overlap between population color volumes defined by two sets of points in color space. The volume from the overlap was then presented relative to: *vsmallest* the volume of the overlap divided by the smallest of that defined by the two input sets of color points and *vboth* the ratio of volume of the overlap and the combined volume of both input sets of color points. Thus, *vsmallest *= 1 indicates that one of the volumes is entirely contained within the other (Maia et al., 2013; Stoddard & Prum, 2008; Stoddard & Stevens, 2011). Additional three colorimetric variables (hue, brightness, and chroma) of the three plumage patches as reviewed in Montgomerie (2006) were calculated using the *summary* function.

Categorical description analysis of the colorimetric variables within FactoMineR package was used to select variables that best described the species per plumage patch at *p* ≤ .05. Subsequently, the selected variables were used in permutational multivariate analysis of variance (with vegan package) using Euclidean distance matrices for partitioning distance matrices among sources of variation and fitting linear models (on species as factors) to distance matrices using a permutation test of 10,000 iterations. All statistical analyses were run using R Statistical Software versions 3.3.1 (R Core Team, 2017).

### 2.7 Genetic sampling and analysis

Fieldwork was performed in Kenya and Tanzania, including Zanzibar as described above, and in Swaziland in March 2015. Tinkerbirds were captured using targeted mist netting with conspecific playback, given a metal and color ring combination, and blood samples were obtained from the ulnar superficial vein (wing) and transferred into 2‐ml cryovials containing 1 ml Queen's lysis buffer (Hobson, Gloutney, & Gibbs, 1997). Samples were stored at −20**°**C in the laboratory, after returning from the field. DNA was extracted using a Qiagen DNeasy blood and tissue kit following the manufacturer's protocols (Qiagen, Valencia, CA, USA). PCR was performed to amplify DNA of the mitochondrial cytochrome *b* gene using primers L14841 (Kocher et al., 1989) and H4a (Harshman, 1994), and nuclear intron β‐fibrinogen 5 using primers FGB5 and FGB6 (Marini & Hackett, 2002) on an Applied Biosystems Thermal Cycler (model 2720) and resulting bands were visualized by gel electrophoresis on a 1% agarose/TAE gel. Cytochrome *b* and β‐fibrinogen intron 5 were sequenced from 64 samples we collected in the field, two samples from Mozambique, obtained from the Muséum National d'Histoire Naturelle (MNHN), and one sample from Mafia Island, Tanzania, obtained from ZMUC. We chose β‐fibrinogen intron 5 because it has been shown to be informative in several avian phylogenetics studies (e.g., Klicka et al., 2014), including in Piciformes (Dufort, 2016; Fuchs & Pons, 2015). Rates of evolution of nuclear genes may vary from those of mtDNA, and we included a nuclear intron because of their slower evolution rate relative to mtDNA (Johnson & Clayton, 2000; Prychitko & Moore, 1997, 2000), they are adaptively neutral due to high substitution rates, with frequent occurrence of indels (insertions and deletions), and they have a lower transition/transversion ratio, with lower homoplasy than mtDNA (Prychitko & Moore, 2000).

The DNA sequences were aligned using ClustalW implemented in MEGA software version 7 (Kumar, Stecher, & Tamura, 2016). For cytochrome *b,* we added outgroup sequences for *Melanerpes carolinus* and *Lybius dubius* (GenBank U89192 and AY279291), as well as three sequences from two *Pogoniulus bilineatus* and one *P. simplex* from a previous study (GenBank MG211663, 64, 70 respectively, Kirschel et al. unpublished ms) and performed analyses on a 1,008‐bp fragment. For β‐fibrinogen intron 5, we also sequenced a sample of *Tricholaema diademata* from Kenya, of *Pogoniulus simplex* from Tanzania, and of *P. subsulphureus* from Nigeria, for use as an outgroup, and performed analyses on a 529‐bp fragment. Newly generated sequences have been deposited in GenBank (MG437418‐79, MG576343‐402).

### 2.8 Phylogenetic analysis

Using our aligned sequences, we compared models of evolution using R package phangorn (Schliep, 2011). We selected the Hasegawa–Kishino–Yano (HKY) substitution model (Hasegawa, Kishino, & Yano, 1985) with invariant distribution for cytochrome *b* (Hasegawa et al., 1985), one of the highest scoring models based on both AICc and BIC scores that had readily available scripts that could be implemented in RevBayes 1.0.3 (Höhna et al., 2016).

The maximum‐likelihood (ML) trees for cytochrome *b* and β‐fibrinogen 5 were constructed using RAxML BlackBox online tool (Stamatakis, 2014; Stamatakis, Hoover, & Rougemont, 2008) using the gamma model of rate heterogeneity for the construction of the ML tree for cytochrome *b* but not β‐fibrinogen 5.

We created a stochastic node with a normal distribution (mean = 8, *SD* = 1, min = 5.38 and max = 11.38) using 5.38 MYA as a minimum age limit for the ancestor of the *Pogoniulus* tinkerbirds (Mlíkovský, 2002), to estimate divergence dates in Revbayes 1.0.5 (Höhna et al., 2016) and applied a single substitution model uniformly to all sites. We avoided having the divergence time reference from being fixed and therefore allowed for flexibility in the calibration of the phylogenetic tree as the divergence may have happened before the proposed 5.38 MYA (Mlíkovský, 2002). We applied birth–death tree priors with the prior mean centered on the expected number of species under a Yule process and a prior standard deviation of 0.587405. This allowed us to create a lognormal distribution with 95% prior probability spanning exactly one order of magnitude within which we fixed our prior uncertainty.

We used a relaxed uncorrelated lognormal clock model and an exponential prior for the mean rate of each partition. The sampling probability was set to the ratio of the tips and estimated total number of described bird species (10426), and we ran a burn‐in phase of Monte Carlo Markov chain (MCMC) sampler for 10,000 iterations under two independent replicates using 13 different moves in a random move schedule with 44 moves per iteration and tuning interval of 250. The main phase of the MCMC analysis was run for one million generations sampling every 200 generations under two independent runs and the same random moves as in the burn‐in phase. Convergence from the independent runs and ESS values were evaluated in Tracer 1.6 (Rambaut, Suchard, Xie, & Drummond, 2014). The final tree was produced from the generated trees by compiling the maximum posteriori tree using a burn‐in of 20%.

### 2.9 Population genetics

We screened our data to determine populations with fixed alleles and removed the uninformative loci from downstream analyses. The strength of our data in discriminating between unique individuals given a random number of loci was determined based on a genotype accumulation curve. The overall quality of our multilocus genotype loci (MLGs) data was examined, including a search for missing data and rare alleles.

The genotypic diversity indices for each of the major (four) populations were estimated: number of multilocus genotypes (MLG) observed as an estimate of genotypic richness, number of expected MLG at the smallest sample size ≥10 based on rarefaction (eMLG), standard error based on eMLG (SE), Shannon–Wiener index of MLG diversity (H, Shannon, 2001), Stoddart and Taylor's index of MLG diversity (G, Stoddart & Taylor, 1988), lambda (Simpson's index; Simpson, 1949), evenness (E.5; Grünwald, Goodwin, Milgroom, & Fry, 2003; Ludwig & Reynolds, 1988; Pielou, 1975), Nei's unbiased gene diversity (H_exp_; Nei, 1978), index of association (*I*_*A*_ Brown, Feldman & Nevo, 1980; Smith, Smith, O'Rourke, & Spratt, 1993), and standardized index of association (r¯d, Agapow & Burt, 2001).

The analysis of the relationship between individuals, subpopulations, and populations was performed using genetic distance measures by calculating the “distance” between samples based on their genetic profile. Provesti's distance (Prevosti, Ocana, & Alonso, 1975) was used in estimating the genetic distances as it returns the fraction of the number of differences between individuals, subpopulations, and populations. These were implemented with the R packages poppr (Kamvar, Tabima, & Grünwald, 2014) and mmod (Winter, 2012). We used the genetic distance matrix to create a neighbor‐joining tree to visualize the relationships in genetic distances at individual, subpopulation, and population levels. To determine whether the major genotypes of the subspecies are closely related and to what degree they contribute to the genotypes of the each other population, we produced a haplotype network (Minimum Spanning Network) for the cytochrome *b* gene using poppr (Kamvar et al., 2014) and ade4 (Dray & Dufour, 2007) R packages. The haplotype network was constructed using 62 sequences (excluding outgroups) of cytochrome *b* that were obtained from 21 different localities.

AMOVA (analysis of molecular variance) was used to detect population differentiation (Excoffier, Smouse, & Quattro, 1992). The AMOVA was implemented in poppr (Kamvar et al., 2014) and ade4 (Dray & Dufour, 2007) R packages based on Provesti's genotypic distance of the cytb gene with the major population levels as the specified strata field (Kenya (coast), Tanzania (coast, including south to Swaziland), Zanzibar, and Udzungwa (and Nguru mountains). Our expectation in a typical panmictic population would be to see most of the variance arising from samples within rather than among populations. We would find evidence that we have population structure in the event that most of the variance occurs among samples between populations.

## 3 RESULTS

**Figure 2:**
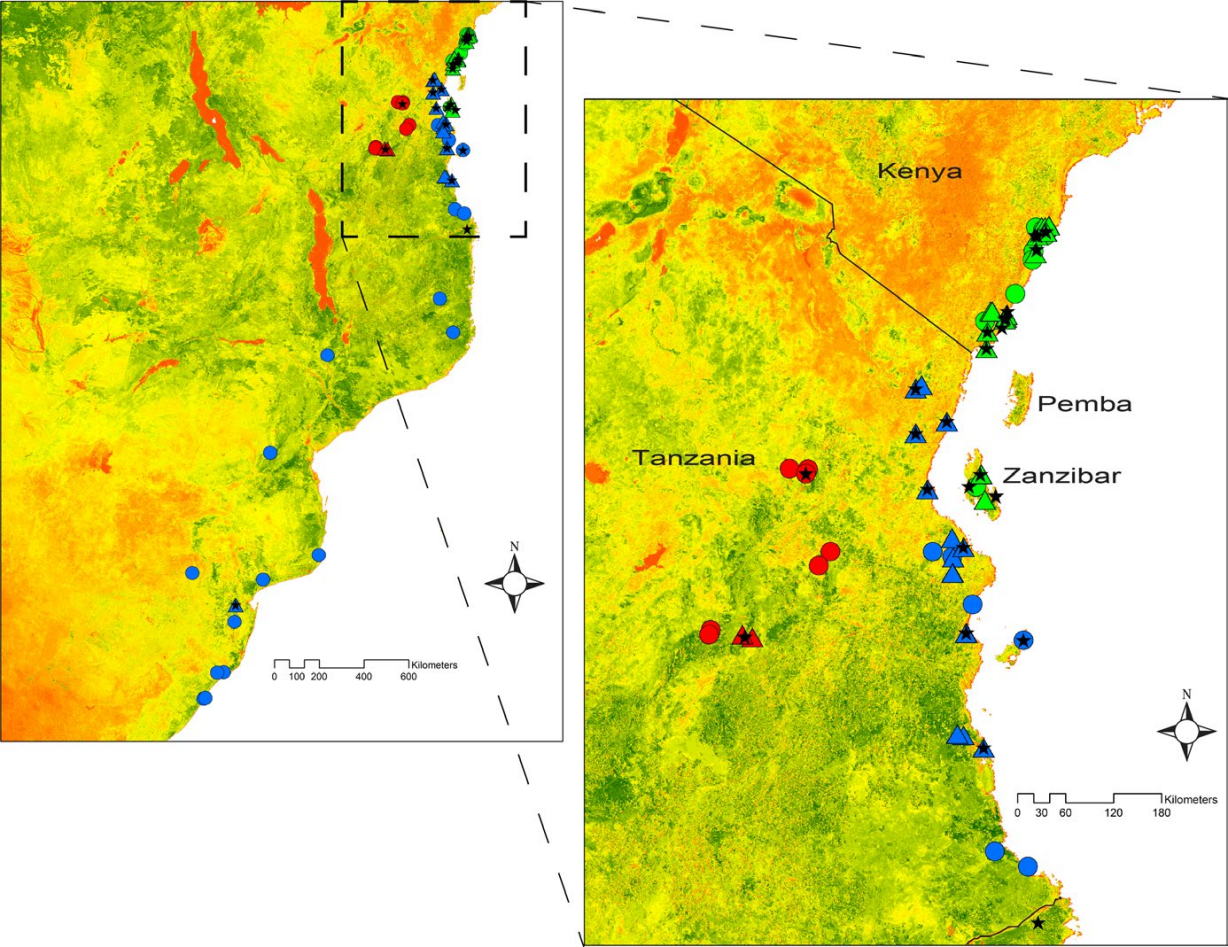
Map of sampling localities showing where recordings (triangles), museum specimens (circles), and genetic material (stars) were obtained. Colors represent the different groups of *fischeri* (green), *bilineatus* (blue), and *conciliator* (red), as analyzed in this study. The inset shows the area in East Africa where the three groups come into close proximity. The background image is based on enhanced vegetation index (EVI) data from the MODIS satellite, with levels of EVI ranging from low (red) to high (dark green)

From our fieldwork performed between 2011 and 2014, we found no evidence of *fischeri* occurring in Coastal Tanzania, where instead we found *bilineatus* (Figure 2). These findings were primarily based on songs recorded in each region, where songs in Coastal Kenya and Zanzibar fit the *fischeri* song type and songs in Tanzania the *bilineatus* song type. All analyses referring to *fischeri* thus refer to populations from Kenya and Zanzibar only.

### 3.1 Song analyses

**Figure 3:**
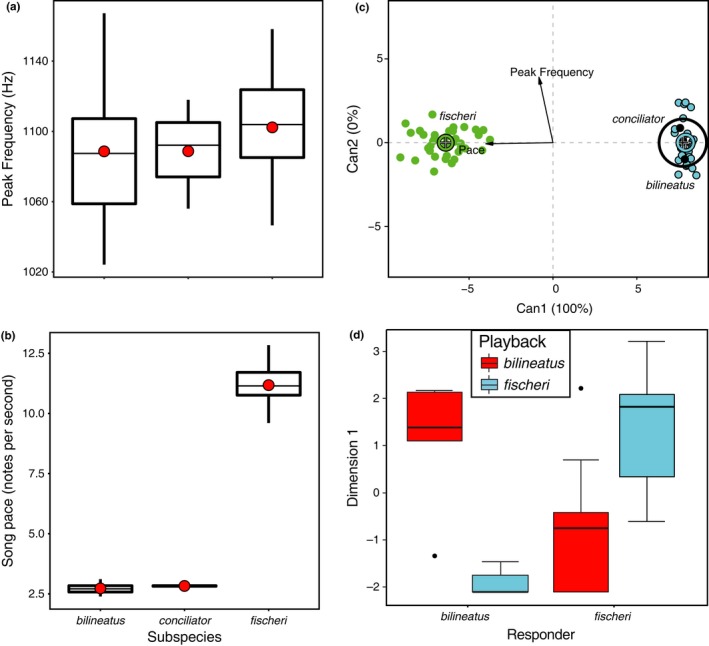
Song traits and responses to playback vary among populations. Boxplots for (a) peak frequency and (b) pace among populations reveal that while there is much overlap in song frequency, *fischeri* sings significantly faster than *bilineatus* and *conciliator*, whose songs do not differ in song pace. (c) Canonical discriminant analysis demonstrates the extent of variation with *fischeri* completely different from *conciliator* and *bilineatus*, which overlap each other. (d) Both *fischeri* and *bilineatus* respond significantly more strongly to their own song than to one another's song, illustrated here with the first principal component dimension

Peak frequency (PF) did not differ among subspecies (*R* ^2^ = 0.0131, *F*_2,64_ = 1.4367, *p* = .245; Figure 3a): with any differences found between *fischeri* (1102.317 ± 8.712 Hz) *bilineatus* (1094.191 ± 6.523 Hz) and *conciliator* (1088.721 ± 21.304 Hz), not significant. Comparison among the populations showed that the insular *fischeri* population on Zanzibar does not sing at a significantly different frequency (1117 ± 11.845 Hz, *F*_1,35_ = 3.025, *p* = .0908) to *fischeri* in Kenya (1097.005 ± 10.265 Hz) and *bilineatus* from Tanzania to Swaziland (1092.449 ± 6.745 Hz). Location (*F*_24,42_ = 1.646, *p* = .0772), latitude (*F*_1,63_ = 0.0004, *p* = .9839) and longitude (*F*_1,63_ = 0.2982, *p* = .5869) had no significant effect on variation in peak frequency.

*P. fischeri* sang significantly faster songs than *bilineatus* and *conciliator* (*F*_2,64_ = 3286.9, *R* ^2^ = 0.99, *p *<* *.0001, Figure 3b). Analyzing for differences in song pace among the populations from Kenya, Tanzania, and Zanzibar revealed a significant difference (*F*_2,64_ = 1711, *R* ^2^ = 0.9842, *p *<* *.0001). Pairwise comparisons revealed significant differences between *bilineatus* from mainland Tanzania and *fischeri* from mainland Kenya (*F*_1,48_ = 5080, *p *<* *.00001), *bilineatus* from mainland Tanzania, and *fischeri* from Zanzibar (*F*_1,33_ = 2735, *p *<* *.00001), but no difference between *fischeri* on Zanzibar and Kenya (*F*_1.35_ = 0.9686, *p* = .3318; Figure S1). We also found no significant difference in song pace between *conciliator* and *bilineatus* (*F*_1,13_ = 0.1572, *p* = .6981). Location (*F*_22,42_ = 1.2456, *p* = .2642), latitude (*F*_1,63_ = 0.0479, *p* = .8275) and longitude (*F*_1,63_ = 0.0021, *p* = .964) had no effect on song pace.

The coefficient of variation was highest in Tanzania for both peak frequency and pace, with pace more variable and frequency less variable in Zanzibar than Kenya (Table 2). The multiresponse permutation procedure of within‐ versus among‐group dissimilarities showed a greater between‐group distance than within‐group distance. Canonical discriminant analysis on peak frequency and song pace clustered *conciliator* with *bilineatus* and retained *fischeri* as distinct (Figure 3c). The discriminant cluster was based on the first dimension which correlates with the song pace (*F*_1,63_ = 303.484, *p *<* *.0001, *R* ^2^ = 99.99), and no discriminate contribution from the peak frequency was observed.

**Table 2:** Coefficient of variation in song rate and peak frequency across regions

Regions	Pace	Peak frequency
Tanzania	6.8504	3.4761
Zanzibar	4.9425	2.1222
Kenya	3.4815	2.5790

### 3.2 Playback experiments

The first dimension produced by the factor analysis of mixed data was significantly correlated with total time, time spent within 10 m, time spent within 20 m, latency, and closest approach distance to the source of the playback stimulus (*R* ^2^ = 65%, *p *<* *.001, Table S2). The second dimension was significantly correlated with preplayback number of songs, postplayback number of songs, and number of songs during playback (*R* ^2^ = 53%, *p* = .007).

The responses of the subspecies during the playback experiment were strongly dependent on the subspecies’ song played (*F*_3,28_ = 10.535, *p* ≤ .001, Figure 3d). Specifically, *bilineatus* responded significantly more strongly to *bilineatus* song (playback stimulus; *F*_1,8_ = 20.766, *p* = .001) than to *fischeri* song, and *fischeri* responded significantly more strongly to *fischeri* song (*F*_1,20_ = 14.319, *p* = .001) than to *bilineatus* song (Figure 3d).

### 3.3 Morphology

PCA was performed with varimax rotation on the morphological data (wing length, tarsus, tail length, bill length, culmen, upper bill depth) from Tanzania coastal region to Southern Africa including Mozambique, Swaziland, Malawi, Zimbabwe, and South Africa (41 specimens), 19 specimens from Kenya mainland and Zanzibar Island and 11 specimens from the Eastern Arc mountain region (Nguru, Uluguru, and Udzungwa). PC1 and PC2 accounted for 43.01 and 18.31 percent variation, respectively (Table S3). PC1 was significantly correlated with wing length, tarsus, tail length, bill length, culmen with *bilineatus* being significantly larger than *fischeri* (*R* ^2^ = 38%, *p *<* *.001, Figure S2, Table S4), and *conciliator* intermediate between the two. Greater body size in *bilineatus* over *conciliator* was mostly attributable to Bergmann's rule effects, with the largest individuals measured coming from populations in higher latitudes (Figure S3). PC2 was significantly correlated with tarsus, culmen, upper bill depth with *fischeri* being larger than *conciliator* (*R* ^2^ = 18%, *p *<* *.001). There was no significant effect of sex on morphology on either PC1 or PC2. Supplementary variables latitude and EVI had a significant positive correlation and longitude a significant negative correlation with PC1. PC2 was significantly negatively correlated with EVI and elevation. VCF had no significant effect on morphology in relation to any of the principal components.

### 3.4 Plumage coloration

#### 3.4.1 Color distance

In terms of chromatic and achromatic distances, *fischeri* was most distinct from the other populations based on just noticeable difference (JND) levels, where JND >2, while most similar were *conciliator* from the Eastern Arc Mts. and *bilineatu*s from Southern Africa (Figure S4). Specifically, for the belly patch, chromatic distances were shorter than achromatic distances (approx. 1–2.3 vs. 2.2–4.5 JND), with largest distances between *fischeri* and *conciliator* followed by *fischeri* and the remaining populations, and smallest between *conciliator* and Southern Africa. In breast plumage, again chromatic distances were shorter than achromatic distances (approx. 1.3–2.1 vs. 2.2–4.7 JND), with largest distances between *fischeri* and *conciliator* and *bilineatus* from Tanzania and *conciliator*, and shortest distances again between *conciliator* and Southern Africa. In contrast, in rump plumage, chromatic distances were greater than achromatic distances (approx. 2.2–4.3 vs. 1.5–3.2 JND), with largest distances between *fischeri* and Southern Africa, and *bilineatus* from Tanzania and Southern Africa, and shortest distances between *conciliator* and Southern Africa and *conciliator* and the remaining populations.

#### 3.4.2 Hue, brightness, and chroma

Visualizing differences in hue based on a plot of color points projected from the tetrahedron to its encapsulating sphere showed that the hue of all plumage patches is between green and red longitudes and specifically in the orange‐yellow region (Figure S5). Analysis of variance based on Euclidean distances revealed significant differences in hue, brightness, and chroma of the three plumage patches (*F*_3,104_ = 3.0736, *p* = .0239, Figure S6). There were significant differences in plumage coloration of the belly patch among populations (*F*_3,27_ = 3.1649, *p* = .0320). Specifically, the highest hue score was found in *conciliator* and *bilineatus* from Tanzania, followed by *fischeri*, and lowest and most distinct in *bilineatus* from Southern Africa. Brightness and chroma, on the other hand, were highest in fischeri, followed by *bilineatus* from Tanzania and Southern Africa, and lowest in *conciliator*. There were also significant differences in mean brightness, hue, and chroma of the breast patch among the populations (*F*_3,27_ = 10.963, *p *<* *.0001); however, in contrast to the belly patch, *bilineatus* from Southern Africa had the highest hue score, indicating a greater contrast in hue across the ventral surface, which was more uniform in *fischeri* and *bilineatus* from Tanzania, and with intermediate contrast in *conciliator*. Breast patch brightness and chroma were higher and similar in *fischeri* and *bilineatus* from Tanzania, and lower and similar in *conciliator* and *bilineatus* from Southern Africa. Moreover, there were significant differences in mean brightness, hue, and chroma of the rump patch among the populations (*F*_3,27_ = 3.164, *p* = .03148) Rump hue was higher and similar in *fischeri*,* bilineatus* from Tanzania, and *conciliator*, and lower and more distinct in Southern Africa *bilineatus*. Rump brightness was highest in Southern Africa and similar in *fischeri*, lower and similar in *conciliator* and *bilineatus* from Tanzania, and rump chroma was again highest in Southern Africa, and distinctly so, and lower and more similar in the other three populations.

#### 3.4.3 Tetracolor plot and model

**Figure 4:**
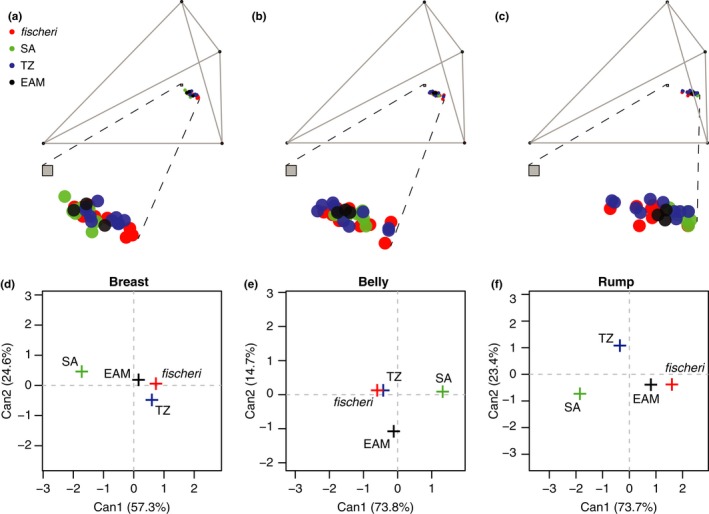
3D plots of tetrahedral color space for the three plumage patches, (a) breast, (b) belly, and (c) rump. Insets illustrate the distribution of points for each population in relation to the achromatic origin (gray square). There is much overlap between *fischeri* (red), *bilineatus* from Southern Africa (SA, green) and from Tanzania (TZ, blue), and *conciliator* from the Eastern Arc Mountains (EAM, black). Canonical discriminant analysis illustrates that at the population level (crosses represent population means), *bilineatus* from Southern Africa is most distant in tetracolor space according to the first two canonical discriminant functions (Can1, Can2) for the plumage patched (d) breast, (e) belly, and (f) rump

Based on the tetracolor space variables (u, s, l, m, h.theta, h.phi, r.vec, r.max, r.achieved) and using multivariate permutational analysis of variance, we found significant differences in the breast (*F*_3,61_ = 2.8122, *p* = .0314) and rump (*F*_3,61_ = 3.9384, *p* = .0119) plumage patches but not the belly (Figure 4a–c, Table S6). Applying canonical discriminant analysis on the tetracolor space variables, we observed that *conciliator* was closer to *fischeri* than to *bilineatus* from Tanzania in breast and rump color. Furthermore, across all plumage patches, we found *bilineatus* from Southern Africa was distinct from *conciliator*,* bilineatus,* and *fischeri* (Figure 4d–f, Table S6).

#### 3.4.4 Color volume overlap

In terms of color volume overlap, *bilineatus* from Southern Africa was the most distinct, with no overlap with conciliator in belly, breast, or rump plumage, no overlap with *bilineatus* from Tanzania in breast (Table S7), low overlap in rump (18%) and belly (40%), no overlap with fischeri in rump, and low overlap in breast (6%) plumage. The greatest extent of color volume overlap was between fischeri and conciliator in rump (93%) and breast (71%), and between *bilineatus* from Tanzania and conciliator (91%) in belly.

### 3.5 Phylogenetic reconstruction

**Figure 5:**
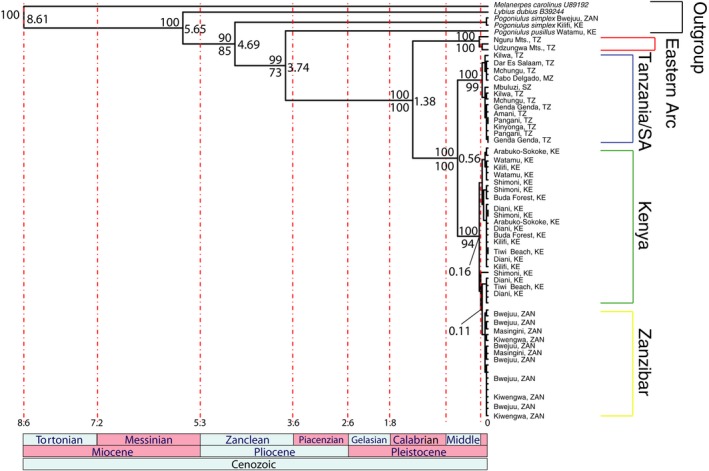
Molecular phylogeny of *P. b. bilineatus, P. b. conciliator,* and *P. b. fischeri* based on the Bayesian inference consensus tree of cytochrome *b*. On the left side of nodes are the posterior probabilities and bootstrap values above and below the branch respectively, with node ages on the right side of the node, calculated using RevBayes 1.0.5. Populations from the Eastern Arc Mountains (*conciliator*) are basal to the rest of the clade, diverging 1.38 mya, while *bilineatus* from Tanzania and South Africa split from the *fischeri* clade 560 kya. The Zanzibar *fischeri* clade split from the Kenya *fischeri* clade 110 kya

We successfully sequenced 62 fragments of 1,008 bp for cytochrome *b* and 56 fragments of 529 bp for β‐fibrinogen 5. Both Bayesian analysis (Figure 5) and maximum‐likelihood analysis (RAxML; Figure S7) of the cytochrome *b* gene revealed that *P. b. fischeri* and *P. b. bilineatus* are reciprocally monophyletic and sister groups, with the insular Zanzibar population monophyletic and nested within the *fischeri* clade from mainland Kenya. The *conciliator* group is basal to *bilineatus* and *fischeri*. Divergence of the subspecies is highly supported based on their maximal Bayesian posterior probability values. In contrast, both Bayesian analysis (Figure S8) and maximum‐likelihood analysis (RAxML; Figure S9) of the more slowly evolving β‐fibrinogen intron 5 showed no evidence of divergence between the populations. No difference was found even between the *conciliator* group and other subspecies in the nuclear intron, in spite of the greater genetic distances found in mitochondrial DNA.

### 3.6 Population genetics

Out of 91 polymorphic loci from the cytochrome *b* gene (62 samples), we removed nine uninformative sites and retained 82 loci for analysis. The data did plateau at 88 sampled multilocus genotype loci necessary to discriminate individuals within populations (Figure S10). The observed number of multilocus genotypes (MLGs) across loci varied between 2 and 3 among individuals.

Comparing populations with the same order of magnitude of samples (thus excluding *conciliator*), genotypic diversity and evenness (H or G) were highest in the Tanzania and Southern Africa *bilineatus* population, followed by Kenya and lower in Zanzibar, although Kenya had the highest genotypic richness based on number of multilocus genotypes (MLG) observed and Nei's unbiased gene diversity (H_exp_), and Zanzibar the lowest (Table S5). Because in our sample the observed MLG was higher than the estimated expected multilocus genotype (eMLG), a more appropriate comparison would be in the eMLG value, which is an approximation of the number of genotypes that would be expected at the largest, shared sample size based on rarefaction. According to eMLG, there is greatest genotypic richness in Tanzania and least in Zanzibar based on populations with sample sizes >10 (Figure S11, Table S5). The same trend was observed in Simpson's index as differences in genotypes among the populations were highest in Tanzania and least in Zanzibar. The distribution of genotype abundances as indicated by evenness (E_5_) suggests that the MLGs observed in Tanzania and Southern Africa population are more evenly spread than in Kenya and Zanzibar.

The individuals showed a structure of clusters into clades that are consistent with their populations (Figures S12 and S13). Greater genetic distance was observed between *conciliator* and other *bilineatus* and *fischeri* populations (Figure S12). High bootstrap values provide significant support for the divergence of the subspecies considered in this study (Figures S13 and S14).

**Figure 6:**
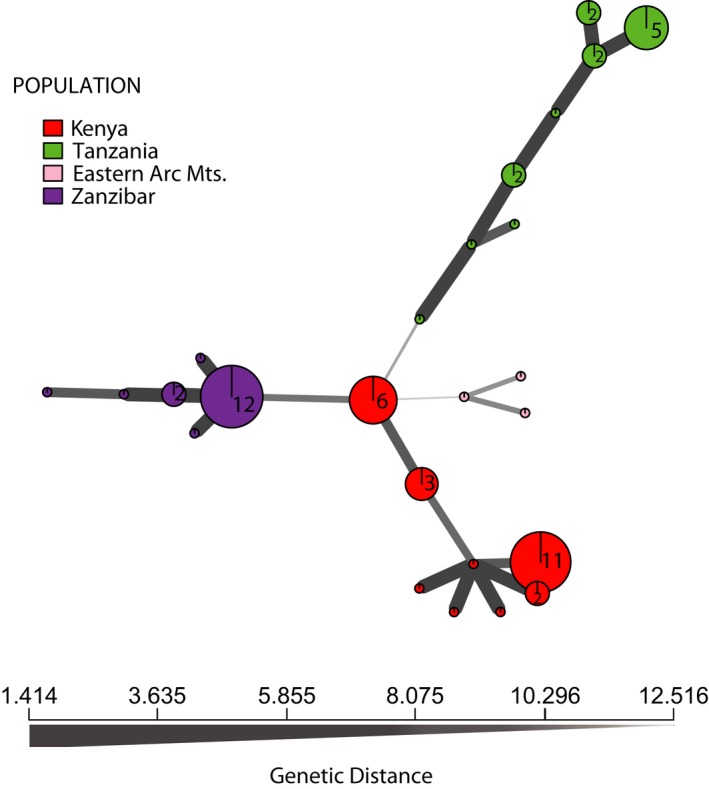
Minimum spanning network using Provesti's genetic distance on 91 polymorphic loci of cytochrome *b* from 62 individuals. Each node represents a unique multilocus genotype. Node shading (colors) represents population membership, while edge widths and shading represent relatedness (genetic distance). Edge length is arbitrary. The network shows a closer genotypic relationship between Zanzibar (violet) and Kenya (red) *fischeri* population relative to *bilineatus* (green) and *conciliator* (pink) populations

The minimum spanning network based on genetic distances showed greater diversity within *fischeri* in Kenya and closer relatedness to *fischeri* from Zanzibar (Figure 6). The present network revealed no connection between *bilineatus* and *conciliator* and between *fischeri* in Zanzibar and *bilineatus* in Tanzania. While Zanzibar and Kenya *fischeri* populations are closely related to one another than with *bilineatus* and *conciliator*, genotypic distances within populations are lower than distances among them (Figure 6).

The variance (σ), the percent of the total variance explained (*R* ^2^), and the population differentiation statistics (ϕ) detected between the populations (σ =* *13.09, ϕ =* *0.909, *R* ^2^ = 90.94, *p* = .001) were greater than within the populations (σ = −0.05, ϕ =* *−0.04, *R* ^2^ = −0.38, *p* = .767) and within samples (σ =* *1.36, ϕ =* *0.905, *R* ^2^ = 9.44, *p* = .001).

## 4 DISCUSSION

Taxonomists have over centuries defined species on the basis of differences in morphology between individuals and/or populations. Yet, the extent of differences that was considered worthy of species rather than subspecies status often remained ambiguous, with overlap in the range of variation found within and among related species (Simpson, 1951). The description of new species and subspecies within the *Pogoniulus* lineage has followed such a path, including the description of *Pogoniulus makawai*, based on a single specimen collected in Zambia in 1964 (Benson & Irwin, 1965), based on, primarily, plumage differences from the local subspecies of *Pogoniulus bilineatus* present at the same locality. Recent phylogenetic studies suggest *P. makawai* is in fact a form of *P. bilineatus* (Corresponding author et al., in review), in spite of the morphological differences found.

Current taxonomy of East African *P. bilineatus* suggests nominate *bilineatus* occurs in Southern Africa and *fischeri* occurs in Eastern Kenya, Tanzania (including the Eastern Arc Mountains), and Zanzibar (Short & Horne, 2002). Our findings in the present study show that the morphological differences thought to delimit species are explained by patterns such as increasing body size with latitude, consistent with Bergmann's rule (Bergmann, 1847), and plumage differences in breast and belly brightness consistent with Gloger's rule (Gloger, 1833), with populations from higher latitudes (Southern Africa) and elevation (Eastern Arc Mountains) less pigmented than those from more tropical climates.

Molecular phylogenetics reveals the evolutionary history of the clade and contradicts many of the morphological findings. While the more slowly evolving nuclear DNA we analyzed (β‐fibrinogen intron 5) does not show evidence of divergence, faster‐evolving mitochondrial DNA (cytochrome *b*) shows three monophyletic lineages, with populations from the Eastern Arc Mountains (*conciliator*) as basal, *P. b. bilineatus* extending from Southern Africa northward and including populations along the coast of Tanzania, and *fischeri* occurring in Kenya and Zanzibar, but apparently absent from coastal Tanzania.

What might have led to the biogeographic pattern of a distinct form in *fischeri* occupying Coastal Kenya and Zanzibar, yet *bilineatus* occurring adjacent to Zanzibar in Tanzania? Might this have resulted from a founder effect, with dispersal from Tanzania to Zanzibar, followed by genetic revolutions in accordance with peripatric speciation (Mayr, 1982), and subsequent dispersal back to the African mainland in Kenya, where *bilineatus* was absent, thus providing the opportunity to establish a population without the presence of a competitor? Our results suggest there is little evidence of peripatric speciation because Zanzibar *fischeri*, although monophyletic, is nested within Kenya *fischeri* (Figure 5, Figure S12). Because dispersal from Kenya to Zanzibar seems unlikely based on geography, we suggest *fischeri* may have been present in Tanzania historically, and certainly during the time of a land bridge connecting the mainland with Zanzibar. Bayesian analysis suggests the Zanzibar population was isolated from Kenya around 110 kya, yet the last common ancestor with *bilineatus* was over 500 kya. With a land bridge present as recently as 10 kya (Prendergast et al., 2016; Rowson, Warren, & Ngereza, 2010), is it possible these two distinct subspecies were interacting so recently? We propose that instead, populations of *fischeri* in coastal Tanzania separated from Zanzibar when sea levels rose at the end of the Pleistocene, and since then have become extinct, following a more recent expansion in the range of *bilineatus*. If this is indeed the case, and *bilineatus* has outcompeted *fischeri* locally in Tanzania, the remaining populations of *fischeri* further north in Kenya could be of conservation concern.

The *fischeri* population from Zanzibar does appear to be diverging genetically and vocally (in song frequency) from Kenya, which we attribute to vicariance, rather than dispersal, following rising Pleistocene sea levels. The absence of tinkerbird species from Pemba Island, which is geographically in between Kenya and Zanzibar and has been isolated from the mainland since at least the Pliocene (Kent, Hunt, & Johnstone, 1971; Rowson et al., 2010), suggests oceanic dispersal is most unlikely in the genus.

But to what extent does genetic isolation reflect reproductive isolation? We found that song rate differences supported the genetic distinction of *fischeri* from *bilineatus*, and confirmed the absence of the former from coastal Tanzania. However, patterns of song variation are discordant with genetic isolation in mtDNA in *conciliator*, which we found overlaps in song characters with *bilineatus*, in spite of its greater genetic distance, with the time of divergence estimated at 1.38 mya. Under the BSC, *bilineatus* could be considered a different species to *fischeri* because of the lack of recognition of its song, whereas it would likely be considered the same species as *conciliator* because their songs are the same. Yet, under the PSC, with *conciliator* basal, it would have to be considered a distinct species if *bilineatus* and *fischeri* were deemed separate species, and hence, we have a taxonomic dilemma regarding how to differentiate species. Acoustic signals play an important role in reproductive isolation (Hoskin, Higgie, McDonald, & Moritz, 2005; Price, 2008; Wilkins, Seddon, & Safran, 2013), and spectral and temporal variations in acoustic signals have been suggested to affect male response and female preference (Gil & Gahr, 2002; Riebel, 2009). Here, rapid divergence has occurred in song in *fischeri*, to the extent that it is unrecognizable to other populations of *bilineatus*, suggesting they would mate assortatively if they coexisted. Meanwhile, other populations evolving in isolation for much longer might interbreed where songs are more similar, evidence for which has been found in other species of *Pogoniulus* (A. Kirschel unpublished data).

The question remains what may have driven such rapid song divergence in *fischeri* when other subspecies of *Pogoniulus bilineatus* that are genetically more isolated all sing the same song (*bilineatus* and *conciliator* presented here, *P. b. leucolaimus* and *P. b. mfumbiri* studied in Kirschel, Blumstein et al., 2009)? We would rule out drift, based on the discordant pattern with genetic distance (Wilkins et al., 2013), and acoustic adaptation to transmission properties or the sound environment (e.g., Kirschel, Blumstein, Cohen et al., 2009, 2011; Slabbekoorn, Ellers, & Smith, 2002; Slabbekoorn & Smith, 2002; Brown and Handford, 1996; Wiley & Richards, 1982), because *fischeri* and *bilineatus* with strikingly different songs occur in similar habitat in the coastal forests of East Africa, while other subspecies of *P. bilineatus* occur in diverse habitats from montane forest to ecotone savanna, yet share the same song. Likewise, arbitrary sexual selection (Prum, 2012) seems unlikely to have driven this rapid divergence, when there's so little song divergence elsewhere in the genus. One possibility is convergence in song with *Pogoniulus simplex*, with which *fischeri* coexists in its entire range. Such convergence might have occurred through introgressive hybridization, though that seems unlikely based on the extent of genetic distance—*P. simplex* is the most basal outgroup within the genus according to both mitochondrial and nuclear DNA analyzed here (Figure 5, Figures S7–S9), or convergent character displacement reducing interference competition (e.g., Grether, Losin, Anderson, & Okamoto, 2009; Grether et al., 2013; Tobias & Seddon, 2009). While previous work on the genus found interference competition led to divergent character displacement (Kirschel, Blumstein et al., 2009), a different interaction based on the extent of ecological competition and relatedness could drive character convergence aiding competitor recognition (Grether et al., 2009).

## 5 CONCLUSION

By integrating genetic analyses with multidimensional phenotypic analyses, we have determined the extent to which the genotype corresponds with the species delimitations of early naturalists and taxonomists, how their delimitations compare with quantitative measurements of morphology and plumage, and how all these characters relate to song, a trait important in reproductive isolation, and one we test recognition of experimentally. While genomic sequencing has transformed the way researchers explore questions in phylogeography and phylogenetics, an integrative approach is still needed to determine the role of variation found in speciation.

## CONFLICT OF INTEREST

None declared.

## AUTHOR CONTRIBUTIONS

ANGK conceived of and designed the study, ANGK and LH performed fieldwork with the help of RKM, AI and ECN performed molecular laboratory work, ANGK, CTP, and ECN analyzed molecular data, ECN and LH analyzed song data, and ECN analyzed plumage data. ECN, CTP, and ANGK wrote the manuscript.
