# Supplementary material for: Rapid song divergence leads to discordance between genetic distance and phenotypic characters important in reproductive isolation
Source: Ecol Evol. 2017 Dec 5;8(1):716–31. doi: 10.1002/ece3.3673 (PMC5756877; doi:10.1002/ece3.3673)
Supplement: Supplementary file 1 [file ECE3-8-716-s001.docx]

**Supplementary Information**

Table S1: Levene’s test of homogeneity of variances across the populations’ song characteristics.

**Song variables Population Mean±Variance df F P**

Song rate *bilineatus* 0.434±1.044e-03

*fischeri* 1.047±1.465e-05 2 2.839 0.0658

*conciliator* 0.451±9.162e-04

Peak frequency *bilineatus* 1088.609±1738.661

*fischeri* 1102.318±613.603 2 2.555 0.0855

*conciliator* 1088.722±968.472


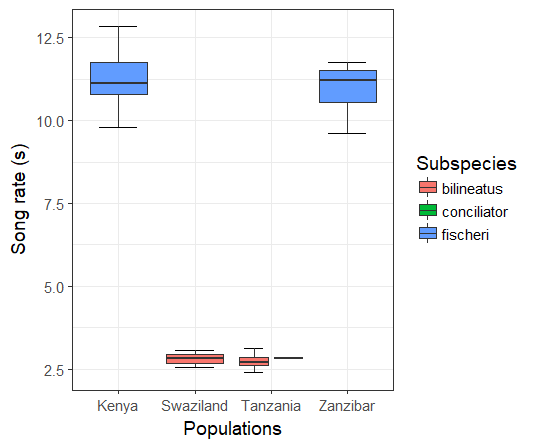


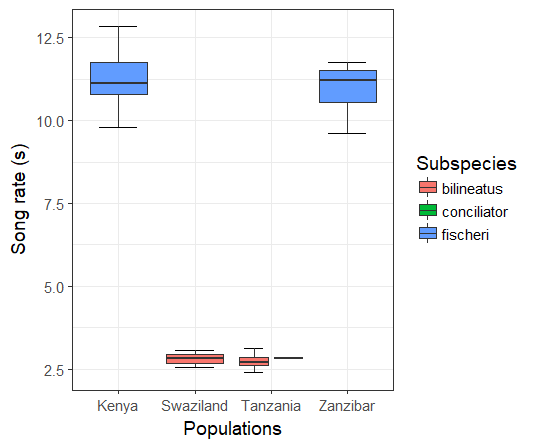

Fig. S1. Song rate by population, showing *fischeri* from Kenya and Zanzibar do not differ in song rate from each other, though sing significantly faster than the other three populations. Southern Africa *bilineatus* (from Swaziland) sing at the same rate as *bilineatus* from Tanzania and *conciliator* from the Eastern Arc Mountains.

Table S2

Principal components extraction on playback measurements. 2 PCs had eigenvalues > 1

-------------------------------------------------------------------------------------

Component Eigenvalue Difference Proportion Cumulative

-------------------------------------------------------------------------------------

Comp1 3.70403 2.50249 0.4630 0.4630

Comp2 1.20154 0.27892 0.1502 0.6132

Comp3 0.922617 0.167815 0.1153 0.7285

Comp4 0.754802 0.191255 0.0944 0.8229

Comp5 0.563547 0.193013 0.0704 0.8933

Comp6 0.370534 0.123263 0.0463 0.9396

Comp7 0.247271 0.011611 0.0309 0.9705

Comp8 0.235659 0.0295 1.0000

---------------------------------------------------------------------------------------

Table S3. Varimax rotation of extracted components based on body size variables.

-----------------------------------------------------------------------------------

Component Variance Difference Proportion Cumulative

------------------------------------------------------------------------------------

Comp1 3.44059 1.97561 0.4301 0.4301

Comp2 1.46498 0.1831 0.6132

---------------------------------------------------------------------------------

Table S4. Rotated Principal Component Matrix using Varimax rotation on morphology variables. -----------------------------------------------------------------------

Variable Comp1 Comp2 Unexplained

-----------------------------------------------------------------------

wing 0.4858 -0.0975 0.2469

tarsus 0.4594 -0.2389 0.359

tail 0.4719 -0.0078 0 .2393

bill_length 0.4019 0.1002 0.3676

culmen 0.2560 0.1720 0.6635

upper_bill -0.1230 0.7063 0.3505

bill_width 0.1151 0.4925 0.512

lower_mandible 0.2735 0.3902 0.3557

------------------------------------------------------------

Fig. S2: Differences in body size between *bilineatus*, *conciliator* and *fischeri* based on the first principal component from the PCA.

Fig. S3: Scatter plot of morphology based on the first two principal components. PC1 was correlated with wing, tarsus, tail and bill length, and PC2 with bill width, upper bill depth and lower mandible length. While there is overlap in PC2 and PC1, in the latter, *fischeri* (red circles) appears smaller overall than *bilineatus*, (blue circles) *and conciliator* (green circles), though less so with the latter after accounting for those individuals at higher latitudes (< -11.0, blue circles with yellow outline), whose greater body size reflects Bergmann’s rule.

Fig. S4: A) Chromatic and B) achromatic distance of the plumage patches of *P. bilineatus* and *P. fischeri*. Boxes represent interquartile range, black lines the median, and red dots the mean values TZ = *bilineatus* from Tanzania, EAM = Eastern Arc Mountains (*conciliator*), SA = *bilineatus* from Southern Africa, *fis* = *fischeri*.

Fig. S5: Hue projection plot of colour points from the plumage patches.

Fig. S6: Boxplots illustrating hue, mean brightness and chroma of belly, breast, and rump patches for four populations. Boxes represent interquartile range, black lines the median, and red dots the mean values. TZ = *bilineatus* from Tanzania, EAM = Eastern Arc Mountains (*conciliator*), SA = *bilineatus* from Southern Africa.

Fig. S7. Maximum likelihood tree (RAxML) of Cytochrome *b*. The branch labels show bootstrap values over 70.

Fig. S8. Bayesian inference consensus tree of β fibrinogen intron 5 (node values represent Posterior values over 0.7).

Fig. S9. Maximum likelihood tree (RAxML) of β fibrinogen intron 5. The branch labels show bootstrap values over 70.

Fig. S10. Genotype accumulation curve for the 91 loci. At 87 the curve plateau suggesting the minimum number of loci required to discriminate individuals across subspecies populations.


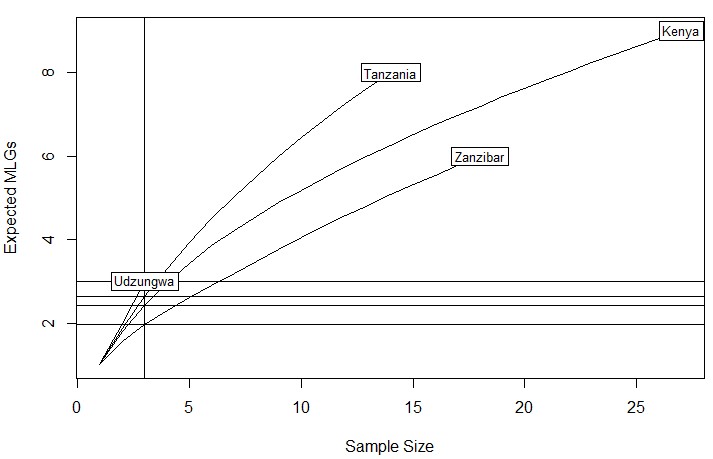
Fig. S11. Rarefaction of the expected genotypic richness based on expected Multi-Locus Genotypes

Table S5: Population genetics summary statistics

| **Population** | **N** | **MLG** | **eMLG** | **SE** | **H** | **G** | **lambda** | **E.5** | **H_exp_** | **I_A_** | **r¯_d_** |
| --- | --- | --- | --- | --- | --- | --- | --- | --- | --- | --- | --- |
| **Kenya** | 27 | 9 | 5.18 | 1.06 | 1.74 | 4.16 | 0.75 | 0.66 | 0.04 | 12.4 | 0.41 |
| **Tanzania** | 14 | 8 | 6.43 | 0.81 | 1.86 | 5.15 | 0.81 | 0.76 | 0.02 | 0.89 | 0.15 |
| **Udzungwa** | 3 | 3 | 3 | 0 | 1.09 | 3 | 0.66 | 1 | 0.08 | -0.63 | -0.06 |
| **Zanzibar** | 18 | 6 | 4.03 | 0.92 | 1.15 | 2.13 | 0.53 | 0.51 | 0.009 | 0.42 | 0.08 |
| **Total** | 62 | 25 | 7.38 | 1.22 | 2.75 | 10.38 | 0.9 | 0.63 | 0.19 | 23.44 | 0.28 |

Abbreviation Statistic

N Number of individuals observed.

MLG Number of multilocus genotypes (MLG) observed.

eMLG The number of expected MLG at the smallest sample size ≥ 10 based on rarefaction

SE Standard error based on eMLG.

H Shannon-Wiener Index of MLG diversity (Shannon, 2001).

G Stoddart and Taylor’s Index of MLG diversity (Stoddart & Taylor, 1988).

lambda Simpson’s Index (Simpson, 1949).

E.5 Evenness, E5 (Pielou, 1975; Ludwig & Reynolds, 1988; Grünwald et al., 2003).

H_exp_ Nei’s unbiased gene diversity (Nei, 1978).

I_A_ The index of association, (Brown, Feldman & Nevo, 1980; Smith et al., 1993).

r¯d The standardized index of association, (Agapow & Burt, 2001).

Fig. S12. Dendrogram based on genetic distance among individuals.

UPGMA tree produced from Provesti’s distance with 10000 bootstrap replicates (node values representing bootstrap values greater than 50% are shown). Edge length is in proportion to the genetic distances.


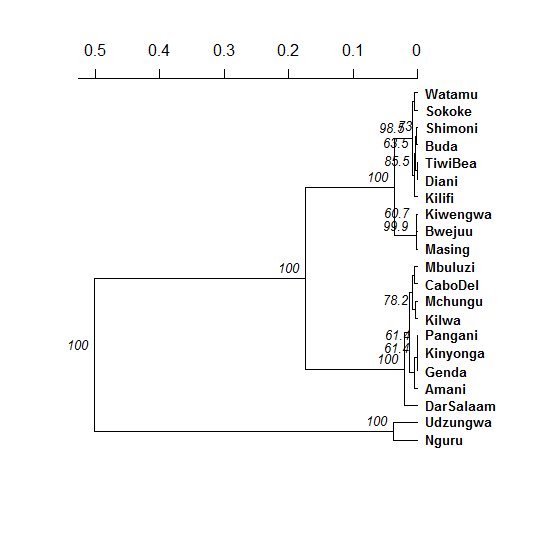


Fig. S13. Dendrogram based on genetic distance among the species sampling locations. UPGMA tree produced from Provesti’s distance with 10000 bootstrap replicates (node values representing bootstrap values greater than 50% are shown).


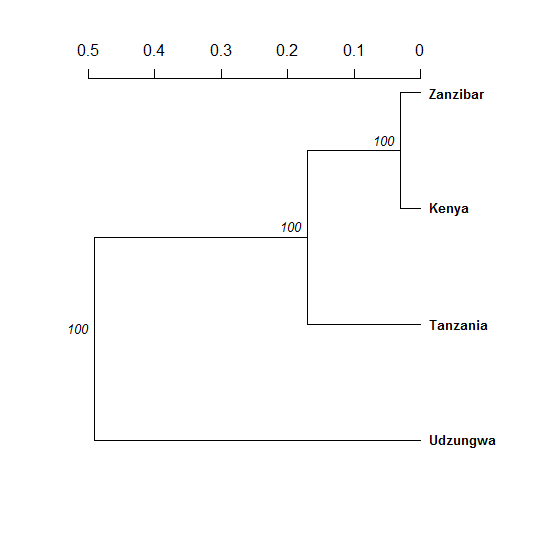


Fig. S14. Dendrogram based on genetic distance among the subspecies populations.

UPGMA tree produced from Provesti’s distance with 10000 bootstrap replicates (node values representing bootstrap values greater than 50% are shown).

Table S6: Coordinates and colorimetric variables that represent reflectance spectra of the populations’ belly, breast and rump patches in the avian tetrahedral color space (mean±sd).

| **Patch** | **Subspecies** | **Violet**  **wavelength** | **Short**  **wavelength** | **medium**  **wavelength** | **Long**  **wavelength** | **h.theta** | **h.phi** | **r.achieved** |
| --- | --- | --- | --- | --- | --- | --- | --- | --- |
| **Belly** | *bilineatus* | 0.13±0.03 | 0.19±0.03 | 0.33±0.02 | 0.33±0.03 | 0.53±0.05 | -0.89±0.11 | 0.47±0.12 |
|  | *conciliator* | 0.13±0.004 | 0.19±0.01 | 0.33±0.01 | 0.32±0.01 | 0.54±0.02 | -0.86±0.04 | 0.44±0.17 |
|  | *fischeri* | 0.12±0.02 | 0.19±0.03 | 0.34±0.02 | 0.34±0.03 | 0.52±0.04 | -0.88±0.09 | 0.51±0.11 |
|  | SA | 0.12±0.01 | 0.18±0.01 | 0.34±0.01 | 0.34±0.01 | 0.49±0.02 | -0.83±0.06 | 0.49±0.04 |
| **Breast** | *bilineatus* | 0.13±0.02 | 0.19±0.01 | 0.33±0.01 | 0.33±0.02 | 0.52±0.02 | -0.88±0.07 | 0.47±0.08 |
|  | *conciliator* | 0.14±0.02 | 0.21±0.01 | 0.32±0.01 | 0.31±0.02 | 0.55±0.04 | -0.97±0.10 | 0.41±0.1 |
|  | *fischeri* | 0.12±0.03 | 0.20±0.02 | 0.33±0.02 | 0.33±0.03 | 0.53±0.05 | -0.93±0.08 | 0.48±0.12 |
|  | SA | 0.15±0.02 | 0.22±0.01 | 0.30±0.01 | 0.31±0.01 | 0.51±0.06 | -1.03±0.07 | 0.38±0.08 |
| **Rump** | *bilineatus* | 0.11±0.02 | 0.09±0.03 | 0.37±0.02 | 0.41±0.03 | 0.43±0.02 | -0.57±0.04 | 0.62±0.14 |
|  | *conciliator* | 0.10±0.01 | 0.08±0.01 | 0.38±0.001 | 0.42±0.01 | 0.41±0.02 | -0.57±0.02 | 0.66±0.03 |
|  | *fischeri* | 0.11±0.01 | 0.10±0.02 | 0.37±0.01 | 0.41±0.02 | 0.40±0.04 | -0.59±0.05 | 0.60±0.08 |
|  | SA | 0.09±0.01 | 0.05±0.01 | 0.39±0.01 | 0.45±0.01 | 0.38±0.02 | -0.53±0.02 | 0.76±0.04 |

Table S7: Colour volume overlap between the subspecies

| **Body part** | **Species1** | **Species2** | **Volume1** | **Volume2** | **Overlap** | **Percentage** |
| --- | --- | --- | --- | --- | --- | --- |
| **Belly** | *bilineatus* | *fischeri* | 9.47e-06 | 1.47e-05 | 6.30e-06 | 66% |
|  | *bilineatus* | *conciliator* | 9.47e-06 | 6.73e-07 | 6.10e-07 | 91% |
|  | *bilineatus* | SA | 9.47e-06 | 8.01e-07 | 3.23e-07 | 40% |
|  | *fischeri* | *conciliator* | 1.47e-05 | 6.73e-07 | 2.91e-07 | 43% |
|  | *fischeri* | SA | 1.47e-05 | 8.01e-07 | 5.25e-07 | 65% |
|  | *conciliator* | SA | 6.73e-07 | 8.01e-07 | 0 | 0% |
| **Breast** | *bilineatus* | *fischeri* | 4.34e-06 | 7.18e-06 | 2.02e-06 | 47% |
|  | *bilineatus* | *conciliator* | 4.34e-06 | 8.21e-07 | 2.84e-07 | 35% |
|  | *bilineatus* | SA | 4.34e-06 | 1.90e-06 | 0 | 0% |
|  | *fischeri* | *conciliator* | 7.18e-06 | 8.21e-07 | 5.85e-07 | 71% |
|  | *fischeri* | SA | 7.18e-06 | 1.90e-06 | 3.34e-07 | 18% |
|  | *conciliator* | SA | 8.21e-07 | 1.90e-06 | 0 | 0% |
| **Rump** | *bilineatus* | *fischeri* | 1.71e-05 | 2.69e-05 | 3.91e-06 | 23% |
|  | *bilineatus* | *conciliator* | 1.71e-05 | 1.11e-06 | 3.92e-07 | 35% |
|  | *bilineatus* | SA | 1.71e-05 | 3.60e-06 | 2.08e-07 | 6% |
|  | *fischeri* | *conciliator* | 2.69e-05 | 1.11e-06 | 1.03e-06 | 93% |
|  | *fischeri* | SA | 2.69e-05 | 3.60e-06 | 0 | 0 |
|  | *conciliator* | SA | 1.11e-06 | 3.60e-06 | 0 | 0 |
